# Anterior Chamber Configuration and Its Related Factors Among 8-Year-Old Children in the Yamanashi Adjunct Study of the Japan Environment and Children’s Study

**DOI:** 10.3390/jcm14155454

**Published:** 2025-08-03

**Authors:** Mingxue Bao, Ryo Harada, Yuka Kasai, Natsuki Okabe, Airi Takahashi, Chio Kuleshov, Yumi Shigemoto, Tadao Ooka, Hiroshi Yokomichi, Kunio Miyake, Reiji Kojima, Ryoji Shinohara, Hideki Yui, Sanae Otawa, Anna Kobayashi, Megumi Kushima, Zentaro Yamagata, Kenji Kashiwagi, on behalf of The Yamanashi Adjunct Study of the Japan Environment and Children’s Study Group

**Affiliations:** 1 Department of Ophthalmology, School of Medicine, University of Yamanashi, Chuo 409-3898, Japan; g23ddm19@yamanashi.ac.jp (M.B.); haradryo@gmail.com (R.H.); kasayuka@yamanashi.ac.jp (Y.K.); nokabe@yamanashi.ac.jp (N.O.); iairi@yamanashi.ac.jp (A.T.); chiok@yamanashi.ac.jp (C.K.); yumishig@yamanashi.ac.jp (Y.S.); 2 Department of Health Sciences, School of Medicine, University of Yamanashi, Chuo 409-3898, Japan; tohoka@yamanashi.ac.jp; 3 Department of Epidemiology and Environmental Medicine, Interdisciplinary Graduate School, Chuo 409-3898, Japan; hyokomichi@yamanashi.ac.jp (H.Y.); kmiyake@yamanashi.ac.jp (K.M.); kojimar@yamanashi.ac.jp (R.K.); 4 Center for Birth Cohort Studies, Interdisciplinary Graduate School of Medicine, University of Yamanashi, Chuo 409-3898, Japan; rshinohara@yamanashi.ac.jp (R.S.); hyui@yamanashi.ac.jp (H.Y.); osanae@yamanashi.ac.jp (S.O.); anna_vfk@yahoo.co.jp (A.K.); kumegumi@yamanashi.ac.jp (M.K.); zenymgt@yamanashi.ac.jp (Z.Y.)

**Keywords:** anterior chamber configuration, Japanese children, axial length

## Abstract

**Objective**: This study aims to examine the anterior chamber structure and related factors in 8-year-old children based on data from The Yamanashi Adjunct Study of the Japan Environment and Children’s Study (JECS). **Methods**: A total of 709 children aged 8 years (350 boys and 359 girls) who participated in the JECS Adjunct Study were included. The right eyes were primarily used for measurements. Optical Coherence Tomography (OCT) was utilized to scan the anterior chambers of the participants’ eyes. The following parameters were measured: Angle Opening Distance (AOD500, 750), Trabecular Iris Space Area (TISA500, 750), Anterior Chamber Angle (ACA500, 750), Peripheral Iris Thickness (IT500, 750), and Peripheral Corneal Thickness (PCT500, 750). The relationships between anterior chamber structure, axial length (AL), spherical equivalent (SE), logMAR (without correction), and body height were analyzed. **Results**: A significant negative correlation was found between SE and ACA (500: coefficient = −0.19; 750: −0.24), AOD (500: −0.19; 750: −0.24), and TISA (500: −0.17; 750: −0.23) (*p* < 0.001). Conversely, a significant positive correlation was observed between AL and ACA (500: 0.22; 750: 0.26), AOD (500: 0.25; 750: 0.30), and TISA (500: 0.24; 750: 0.29) (*p* < 0.001). Boys exhibited a longer AL (boys: girls = 23.30 ± 0.76 mm; girls = 22.79 ± 0.72 mm) and greater CT (500: boys = 812.82 ± 51.94 mm; girls = 784.48 ± 51.81 mm; 750: boys = 776.01 ± 48.64 mm; girls = 751.34 ± 49.63 mm) compared to girls (*p* < 0.001) despite no significant difference in body height. CT and IT showed no correlation with AL or SE, and visual acuity had minimal correlation with IT and CT. **Conclusions**: In our cohort of eight-year-old children, the anterior chamber angle structure correlates with ocular structures and refractive error, revealing notable differences between boys and girls.

## 1. Introduction

Glaucoma is the leading cause of irreversible blindness worldwide [1]. In Japan, the prevalence of glaucoma is reported to be 5% among individuals aged 40 and older. Among all types of glaucoma, primary open-angle glaucoma (POAG) is the most common, with a prevalence of 3.9%, while primary angle-closure glaucoma (PACG) has a prevalence of only 0.6% [2,3]. PACG is particularly prevalent in certain Asian regions and can lead to blindness if not treated promptly [4]; thus, early detection is crucial. The angle configuration plays an important role in regulating intraocular pressure and the development of glaucoma. Previous studies on angle configuration primarily involved adults, revealing correlations between angle closure and factors such as age and gender [5,6,7,8]. However, limited research has focused on children due to their lack of cooperation during gonioscopy, which is uncomfortable and invasive. Recent advancements in optical coherence tomography (OCT) have enabled the examination of angle configuration in children [9]. One study reported that the width of the anterior chamber and the depth of the peripheral anterior chamber increase from birth to around 5 years of age, while another study found that angle openness increases with growth [10]. Additionally, a relationship between angle configuration and hyperopia has been noted [11]. Despite these insights, investigations into the associations between anterior chamber configuration and factors such as refractive error and axial length in children—particularly in East Asian populations—remain limited. Further research is needed to identify the underlying determinants and to establish normative reference values for ocular development in childhood.

## 2. Subjects and Methods

Since 2011, the Ministry of the Environment (MOE) has been conducting the Japan Environment and Children’s Study (JECS). The JECS is a prospective birth cohort study that aims to elucidate how the environment affects children’s health and create an environment in which children can grow up healthy [12,13]. Approximately 4400 participants participated in The Yamanashi Adjunct Study of the JECS. The protocol has been conducted in various medical fields, including ophthalmology, pediatrics, obstetrics and gynecology, orthopedics, otolaryngology, endocrinology, dentistry, oral surgery, immunology, and others. Ophthalmologic examinations encompass assessments of visual acuity, refractive error (expressed as the spherical equivalent -SE), axial length, OCT examinations, as well as questionnaires for caregivers. Building upon the findings from these studies, this research aims to investigate the correlation between angle configuration parameters, refractive error, axial length, and visual acuity in children. The primary objective of this study is to elucidate the factors influencing the angle configuration.

The study will proceed with the prior approval of the Steering Committee Chair and the Director of the Program Office; written informed consent was obtained from all participants. Following the principles outlined in the Declaration of Helsinki. The JECS protocol was reviewed and approved by the Ministry of the Environment’s Institutional Review Board on Epidemiological Studies (IRB number: 100910001). The study was approved by the Institutional Review Board of Yamanashi University (No. 2070).

The study targets eight-year-old participants who took part in the JECS-Y (Japan Environment and Children’s Study—Yamanashi) conducted at the University of Yamanashi in April 1 2021 through March 31 2023. The participants underwent an eye examination, a body composition test, and a questionnaire survey. All examinations were conducted on the same day. The ophthalmological examination included the following parameters: uncorrected visual acuity, corrected visual acuity, spherical equivalent (SE) without the use of a cycloplegic agent, axial length (AL), and anterior segment optical coherence tomography (OCT). All ophthalmologic examinations were performed by ophthalmologists and orthoptists. Visual acuity was measured using an autorefractor (NIDEK AR-F, Gamagori, Japan). If visual acuity was found to be less than 20/20, corrected visual acuity was measured based on refractive values without the use of an accommodation paralytic agent. AL measurements were conducted for both eyes using an optical measuring device (NIDEK AL-SCAN, Gamagori, Japan). The quantification of the angle chamber measurements was performed using the anterior segment mode and angle mode of OCT (NIDEX EX, Gamagori, Japan). This device enables rapid image acquisition without direct contact with the eyeball. During the imaging process, alignment was ensured by utilizing a fixed lamp in the OCT. Multiple scans were performed, and the image with the highest clarity was selected for analysis. To ensure image quality, a signal strength index (SSI) threshold of 7 or higher was applied. Cases that presented difficulties in imaging due to non-cooperation from the subject, inadequate fixation, improper positioning of the head or forehead, as well as images with an SSI below 7, were excluded from the analysis. Subsequently, the corner angle measurement mode of NAVIS-EX was employed for the angle measurements. To ensure recognition accuracy, the position of the scleral spur was manually identified by an examiner according to the criteria [8,14,15].

**Figure 1:**
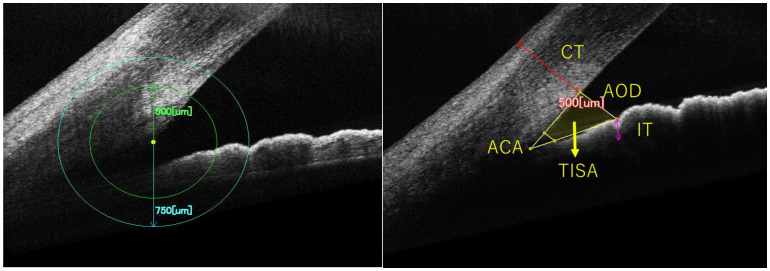
Definition of Anterior Chamber Angle Configuration Parameters Using OCT: A Right Eye Example. ACA, AOD, TISA, PIT, PCT were measured at 500 μm and 750 μm from the scleral spur. ACA: the anterior chamber angle, AOD: angle opening distance, TISA: trabecular iris space area, IT: iris thickness, CT: corneal thickness.

The measured parameters, including the anterior chamber angle (ACA), angle opening distance (AOD), trabecular iris space area (TISA), peripheral iris thickness (PIT), and peripheral corneal thickness (PCT), were determined using established methodologies reported in previous studies [16,17,18]. Anterior chamber angle parameters were measured in both eyes of all participants, excluding images of insufficient quality due to inadequate cooperation. Measurements were conducted at 500 µm and 750 µm from the scleral spur, with five parameters assessed at each location. The AOD500 (750) was the distance from the scleral spur on the posterior surface of the cornea to the anterior surface of the iris, measured along a perpendicular line drawn from the corneal endothelial surface at a position of 500 (750). TISA500 (750) denotes the area enclosed by a line drawn from the scleral spur to the iris surface, parallel to AOD500 (750). The ACA500 (750) is defined as the angle formed between the posterior corneal surface and the iris surface at 500 (750) from the scleral spur. IT500 (750) represents the thickness of the iris at the location corresponding to AOD500 (750), while CT500 (750) signifies the corneal thickness at the same AOD500 (750) position (Figure 1).

### Statistical Analysis

The statistical analysis in this study was conducted using Easy R software (Mac OS X version). LogMAR was used to evaluate visual acuity. Associations between anterior chamber angle parameters and AL, SE, uncorrected logMAR visual acuity, and height were analyzed. Differences in the logMAR visual acuity, AL, SE, anterior chamber angle parameters, and body measurements between sexes were assessed using independent t-tests. The degree of association between the variables was assessed using Pearson’s product–moment correlation coefficient. Multivariable regression analysis was conducted using anterior chamber angle parameters as dependent variables, with AL, SE, uncorrected logMAR visual acuity, sex, and height as independent variables. Mean values and 95% confidence intervals (CI) were reported for each parameter, and a significance level of 5% was adopted as the threshold for statistical significance. Numbers are expressed as mean ± standard deviation.

## 3. Results

### 3.1. Participants

Excluding 207 individuals who did not meet the eligibility criteria among a total of 916 applicants, the exclusions included 54 patients who did not cooperate with anterior ocular (OCT) imaging, 116 patients who had difficulty undergoing OCT imaging according to the enrollment criteria, and 37 patients whose visual acuity measurement showed poor reliability. Of the 709 applicants who met the enrollment criteria, 350 were boys and 359 were girls. In total, 460 of the right eyes were employed and 249 of the left eyes were employed when the right eye did not meet the enrollment criteria. The mean SSI analyzed was 9 ± 1 (range 7–10).

### 3.2. Correlation of Angle Parameters with SE, AL, and logMAR

**Figure 2:**
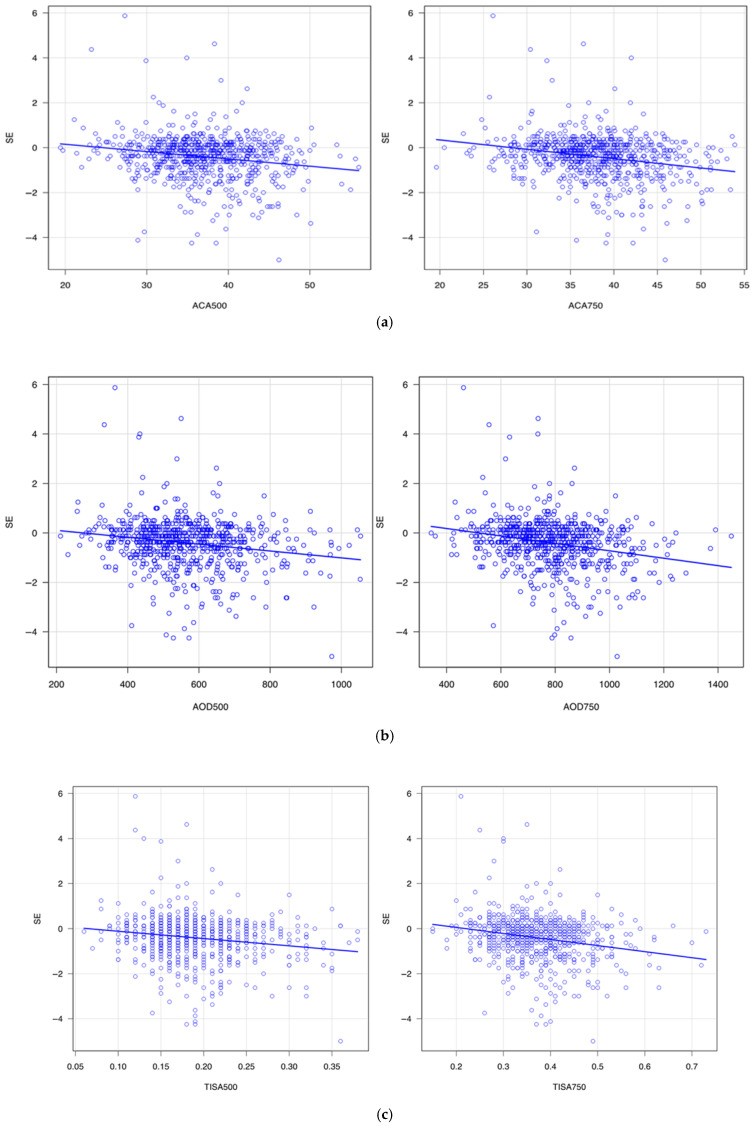
(**a**). Correlation between spherical equivalent (SE) and anterior chamber angle (ACA). (**b**). Correlation between spherical equivalent (SE) and angle opening distance (AOD). (**c**). Correlation between spherical equivalent (SE) and trabecular iris space area (TISA).

**Figure 3:**
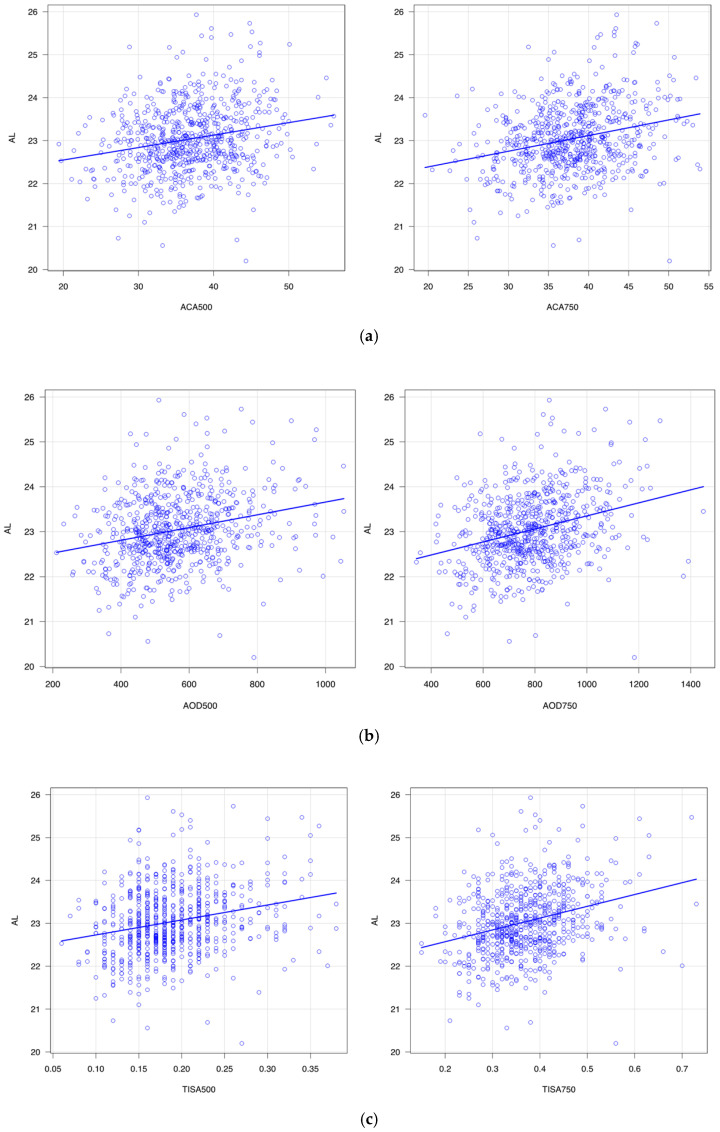
(**a**). Correlation between axial length (AL) and anterior chamber angle (ACA). (**b**). Correlation between axial length (AL) and angle opening distance (AOD). (**c**). Correlation between axial length (AL) and trabecular iris space area (TISA).

**Figure 4:**
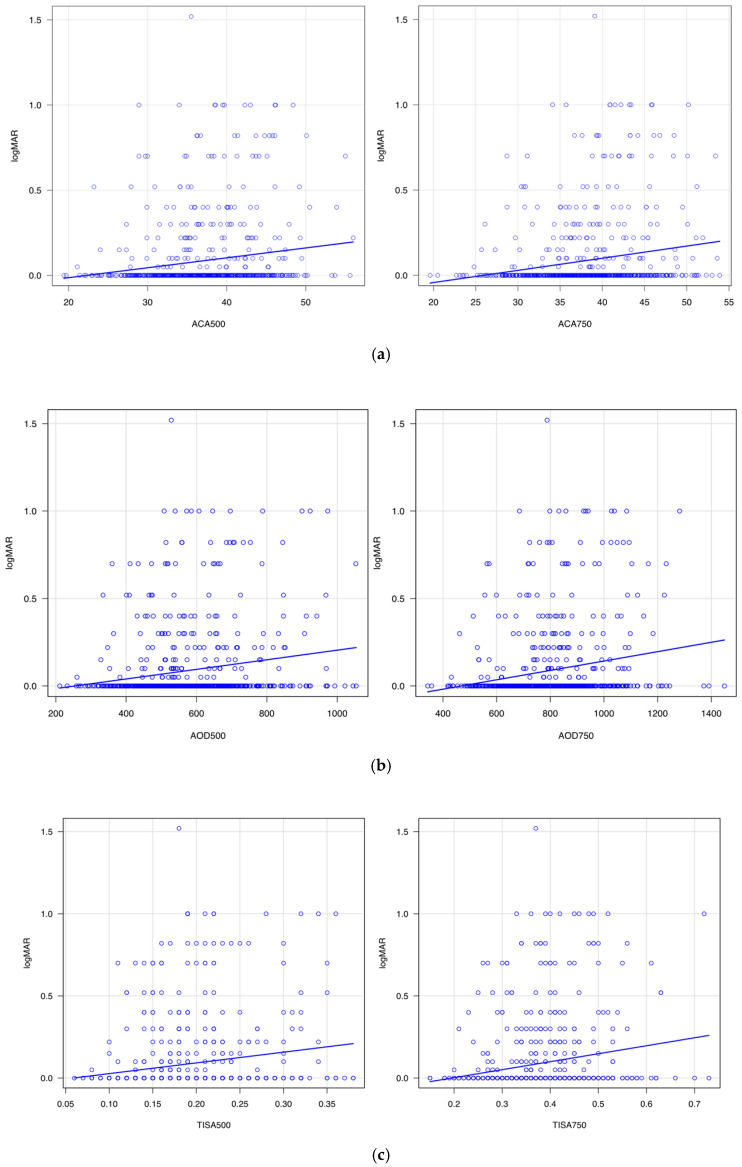
(**a**). Correlation between logMAR and anterior chamber angle (ACA). (**b**). Correlation between logMAR and angle opening distance (AOD). (**c**). Correlation between logMAR and trabecular iris space area (TISA).

Table 1 summarizes the correlation of angle configuration with SE, AL, and uncorrected LogMAR. Figure 2,Figure 3 and Figure 4 show scatter plots of their correlations. ACA500 (*r* = −0.19, *p* < 0.001), ACA750 (*r* = −0.24, *p* < 0.001), AOD500 (*r* = −0.19, *p* < 0.001), AOD750 (*r* = −0.24, *p* < 0.001), TISA500 (*r* = −0.17, *p* < 0.001), and TISA750 (*r* = −0.23, *p* < 0.001) showed significant negative correlations with SE. In contrast, ACA500 (*r* = 0.22, *p* < 0.001), ACA750 (*r* = 0.26, *p* < 0.001), AOD500 (*r* = 0.25, *p* < 0.001), AOD750 (*r* = 0.30, *p* < 0.001), TISA500 (*r* = 0.24, *p* < 0.001), and TISA750 (*r* = 0.29, *p* < 0.001) exhibited significant positive correlations with AL. Moreover, ACA500 (*r* = 0.16, *p* < 0.001), ACA750 (*r* = 0.19, *p* < 0.001), AOD500 (*r* = 0.18, *p* < 0.001), AOD750 (*r* = 0.21, *p* < 0.001), TISA500 (*r* = 0.16, *p* < 0.001), and TISA750 (*r* = 0.19, *p* < 0.001) were positively correlated with uncorrected logMAR visual acuity. PCT500 (*r* = 0.11, *p* = 0.004) and PCT750 (*r* = 0.11, *p* = 0.006) demonstrated a positive correlation with AL, whereas no significant correlation with SE or uncorrected logMAR was observed. In addition, PIT (500, 750) showed no significant correlation with SE or uncorrected logMAR at either the 500 μm or 750 μm distances.

Figure 2 shows the correlation of SE and ACA (Figure 2a), AOD (Figure 2b), and TISA (Figure 2c). These three angle parameters became greater with the myopia shift in refractive error. Figure 3 shows the correlation of the angle parameters with AL. The angle parameters became greater with the elongation of AL. Figure 4 shows the correlation of angle parameters with uncorrected logMAR. The angle parameters became greater with the deterioration of uncorrected logMAR.

### 3.3. Comparison Between Boys and Girls

Table 2 summarizes the differences between boys and girls. There were no significant differences between boys and girls in terms of SE, uncorrected logMAR, and body height; however, boys showed a significantly longer AL compared to girls (boys: 23.30 ± 0.76 mm; girls: 22.79 ± 0.72 mm) (*p* < 0.001). Several angle parameters showed significant differences between boys and girls. Boys had significantly greater PCT500 and PCT750 values than girls (PCT500: boys = 812.82 ± 51.94 µm, girls = 784.48 ± 51.81 µm; PCT750: boys = 776.01 ± 48.64 µm, girls = 751.34 ± 49.63 µm; *p* < 0.001). In addition, boys exhibited significantly wider AOD750 (*p* = 0.02) and ACA750 (*p* = 0.007) compared to girls. TISA750 also tended to be larger in boys, although the difference did not reach statistical significance. No significant sex-related differences were observed in SE, uncorrected logMAR visual acuity, or height.

### 3.4. Multiple Regression Analysis of Factors Influencing the Angle Configuration

Table 3 shows the results of the multiple regression analysis with angle configuration parameters as the dependent variables, and AL, SE, uncorrected logMAR, gender, and height as the explanatory variables. ACA500 (*β* = 0.13, *p* < 0.001), ACA750 (*β* = 0.15, *p* < 0.001), AOD500 (*β* = 0.18, *p* < 0.001), AOD750 (*β* = 0.20, *p* < 0.001), TISA500 (*β* = 0.18, *p* < 0.001), and TISA750 (β = 0.22, *p* < 0.001) showed significant positive associations with axial length (AL). In contrast, ACA500 (*β* = −0.11, *p* = 0.005), ACA750 (*β* = −0.14, *p* < 0.001), AOD500 (*β* = −0.08, *p* = 0.009), AOD750 (*β* = −0.12, *p* < 0.001), TISA500 (*β* = −0.06, *p* = 0.03), and TISA750 (*β* = −0.10, *p* = 0.003) were negatively associated with spherical equivalent (SE). Additionally, AOD500 (*β* = 0.10, *p* = 0.008), AOD750 (*β* = 0.07, *p* = 0.04), TISA500 (*β* = 0.10, *p* = 0.005), and TISA750 (*β* = 0.08, *p* = 0.03) showed significant positive correlations with body height at both measurement distances. Only the CT of boys was significantly thicker than that of girls at both the 500 μm (*β* = −0.24, *p* < 0.001) and 750 μm (*β* = −0.20, *p* < 0.001) distances. There was no significant correlation between IT and CT with all explanatory variables except for gender. The uncorrected logMAR did not show any significant correlation with all investigated parameters.

## 4. Discussion

Angle configuration not only contributes to the regulation of intraocular pressure and the development of glaucoma. Therefore, it is crucial to investigate the factors involved in angle configuration. As stated in the review article by Chen et al. [19], previous studies on pediatric eyes have primarily focused on measurements such as AL, central CT, the central anterior chamber depth, and lens thickness [11,20,21,22]. However, there is a limited amount of analysis on the angle configuration associated with the development of ocular morphology and function in pediatric eyes [19]. Table 4 shows previous studies on pediatric ocular structures [11,20,21,22]. In this study, we examined the anterior configuration and the associated factors employing a larger sample size compared to previous reports. As a result, it was found that there is a significant relationship between the anterior configuration and AL, SE, and body height according to the multiple regression analysis. Additionally, we found a significant gender difference in several angle configuration parameters.

Shimizu et al. reported that AOD500 is correlated with AL and SE [23] by studying 154 participants aged 6 months to 15 years, after adjusting for the age effect. Edawaji et al. reported that AOD and TISA showed a negative correlation (*p* < 0.001) with SE [11]. Daniel et al. reported nasal angle configuration parameters in 50 participants aged 4 to 16 years, in which AOD500, AOD750, and TISA750 showed a significant negative correlation with SE. These results were consistent with the current findings. However, the correlation between SE and TISA500 was not consistent with a previous study [20]. Amerasinghe et al. reported that AOD500 and TISA500 exhibit a negative correlation with SE and a positive correlation with AL [24], which is consistent with the current study. The possible reasons for this discrepancy include differences in sample size, ethnic variations, and variations in measurement devices. Although no previous studies investigated the correlation of angle configuration with IT among children, the current findings are consistent with previous reports in adults [25,26]. In a study involving 86 adult patients with angle-closure and open-angle glaucoma, the univariate analysis revealed a significant positive correlation between AOD500 and AL [26]. The correlation between AL and angle configuration parameters was observed in normal eyes as well as eyes with angle-closure or open-angle glaucoma, indicating the importance of eye development in determining angle configuration.

It is well-established that there are gender differences in the axial length of the eye [10,27,28,29]. This study revealed that boys have a significantly larger AL compared with girls despite having a similar body height, as reported in our previous report [27]. In addition to AL, some angle configuration parameters show a significant gender difference. Previous papers reported gender differences in ACA, AOD, and TISA among children aged from 2 days to 15 years and 52 adults in 20 s [11,30]. Amerasinghe et al. reported significant gender differences in AOD500 and nasal TISA500 among adults at 50 s [24]. This gender difference could potentially be influenced by age, lens status, and race. Gender differences in angle configuration among children may indicate that gender influences the development of angle configuration. We only included 8-year-old children in the current study. The significant gender difference observed in this study may suggest a possible association with a higher prevalence of angle-closure glaucoma in females. It is necessary to investigate gender differences in the development of angles using a wide age range or a longitudinal study. Zheng et al. reported that central and 3.0 mm peripheral corneal thickness is significantly thinner in females compared to males [31], which is consistent with the current result. Although the current result did not show a significant gender difference, it is an important factor in angle-closure glaucoma, and Mak et al. reported that the average iris volume, along with open-angle and angle-closure iris volumes, is a significant determinant of the anterior chamber angle [32]. Therefore, it is necessary to investigate the physiological or pathological implications of such gender differences in these factors further.

The current study has some limitations. Although the examinations were scheduled to occur as close as possible to the month in which participants turned 8 years of age, they were primarily conducted during holidays and vacation periods to improve participation rates. As a result, the assessments were not strictly performed at 8 years and 0 months. However, since the examinations were conducted at ages closely approximating 8 years, any potential developmental differences are unlikely to have significantly influenced the study findings. Refractive error was conducted without using accommodative paralysis agents, which introduces the possibility of deviation from the true values in the measured SE. Due to instrumental limitations, OCT evaluation was only performed on the temporal side. Pinilla et al. reported that angle parameters such as ACA and AOD are larger on the nasal side compared to the temporal side [30]. It is necessary to measure other portions in addition to the temporal side in future studies. Approximately 22.6% of all subjects were excluded by satisfying exclusion criteria. Thus, there is a possibility of selection bias in the study population. A comparison between the cases included in the analysis and those excluded in this study revealed no significant differences in the sex ratio, uncorrected visual acuity, axial length, or refractive error. Therefore, the present findings may be considered representative of the anterior chamber angle configuration in Japanese 8-year-old children. We failed to evaluate some important factors related to angle closure glaucoma, such as the lens vault [33], lens thickness, and iris curvature in the current study. Given the need to conduct a wide range of multidisciplinary assessments within a limited timeframe, it was not feasible to allocate additional time for the measurement of these parameters. These parameters should be subject to future investigations. Chansangpetch et al. reported that there are significant racial differences in anterior angle parameters such as AOD500, AOD750, TISA500, and TISA750 among adult subjects [34]. It is well known that there is a significant racial difference in the prevalence of angle-closure glaucoma among adult subjects. There are few studies on racial differences in angle configuration among children. Further studies in this area are necessary.

According to population-based surveys in East Asia and Southeast Asia, the prevalence of primary angle-closure glaucoma has consistently been high [35]. The results of this study may provide fundamental data on angle morphology in children and contribute valuable insights for research on angle-related conditions such as angle closure. We are now conducting long-term prospective studies to investigate the longitudinal changes in angle configuration and associated factors. Additionally, this study incorporates the analysis of prenatal and postnatal lifestyle habits, environmental factors, as well as various physical and mental information beyond the scope of ophthalmology. These upcoming studies may elucidate the relationship between changes in angle configuration over time, their influencing factors, and the structural changes in the eye, and how they relate to the occurrence of future eye diseases. The conclusions of this article are solely the responsibility of the authors and do not represent the official views of the above government.

## Figures and Tables

**Table 1:** Correlation of the angle parameters with SE, AL, and logMAR.

Parameters	SE (Correlation Coefficient, *p*-Value)	AL (Correlation Coefficient, *p*-Value)	Uncorrected logMAR (Correlation Coefficient, *p*-Value)
**ACA500**_μm_	−0.19 (<0.001)	0.22 (<0.001)	0.16 (<0.001)
**ACA750**_μm_	−0.24 (<0.001)	0.26 (<0.001)	0.19 (<0.001)
**AOD500**_μm_	−0.19 (<0.001)	0.25 (<0.001)	0.18 (<0.001)
**AOD750**_μm_	−0.24 (<0.001)	0.30 (<0.001)	0.21 (<0.001)
**TISA500**_μm_	−0.17 (<0.001)	0.24 (<0.001)	0.16 (<0.001)
**TISA750**_μm_	−0.23 (<0.001)	0.29 (<0.001)	0.19 (<0.001)
**PCT500**_μm_	0.05 (0.22)	0.11 (0.004)	−0.06 (0.12)
**PCT750**_μm_	0.08 (0.04)	0.11 (0.006)	−0.08 (0.04)
**PIT500**_μm_	−0.01 (0.76)	0.05 (0.21)	−0.03 (0.41)
**PIT750**_μm_	−0.01 (0.83)	0.04 (0.35)	−0.02 (0.55)

**Table 2:** Comparison of the parameters investigated between the boys and girls.

	Boy (*n* = 350) Mean ± SD (95%CI)		Girl (*n* = 359) Mean ± SD (95%CI)		*p*-Value (Boys vs. Girls)
**ACA500**_μm_	37.36 ± 6.07	19.40–55.60	36.51 ± 5.73	22.20–56.00	0.06
**AOD500**_μm_	569.76 ± 143.87	211.00–1.54.00	559.56–133.24	258.00–993.00	0.33
**TISA500**_μm_	0.19 ± 0.06	0.06–0.38	0.19 ± 0.05	0.08–0.37	0.53
**PIT500**_μm_	248.31 ± 42.15	121.00–245.00	242.65 ± 39.11	139.00–391.00	0.06
**PCT500**_μm_	812.82 ± 51.94	686.00–977.00	784.48 ± 51.80	658.00–1072.00	<0.001
**ACA750**_μm_	38.46 ± 5.90	19.60–53.90	37.31 ± 5.37	20.50–51.20	0.007
**AOD750**_μm_	800.94 ± 173.98	358.00–1449.00	772.47 ± 148.53	343.00–1372.00	0.02
**TISA750**_μm_	0.38 ± 0.09	0.15–0.73	0.36 ± 0.08	0.15–0.70	0.06
**PIT750**_μm_	287.05 ± 43.76	150.00–422.00	282.05 ± 42.85	156.00–419.00	0.13
**PCT750**_μm_	776.01 ± 48.64	631.00–954.00	751.34 ± 49.63	634.00–1028.00	<0.001
**AL (mm)**	23.30 ± 0.76	21.32–25.93	22.79 ± 0.72	20.20–25.61	<0.001
**SE (D)**	−0.40 ± 0.96	−5.00–+4.38	−0.41 ± 1.04	−4.25–+5.88	0.86
**logMAR**	0.08 ± 0.20	0.00–1.00	0.09 ± 0.22	0.00–1.52	0.41
**Height (cm)**	125.44 ± 4.88	113.40–139.50	124.99 ± 4.88	113.20–142.50	0.22

**Table 3:** Multivariable regression analysis of the associations between anterior chamber angle configuration parameters and axial length, refractive error (spherical equivalent), sex, and height. Values are expressed as standardized regression coefficients (*β*).

Response Variable	AL		Height		logMAR		SE		Gender	
	*β*	*p*	*β*	*p*	*β*	*p*	*β*	*p*	*β*	*p*
**ACA500**_μm_	0.13	<0.001	0.06	0.11	0.05	0.30	−0.11	0.005	−0.03	0.45
**ACA750**_μm_	0.15	<0.001	0.05	0.20	0.05	0.22	−0.14	<0.001	−0.05	0.23
**AOD500**_μm_	0.18	<0.001	0.10	0.008	0.06	0.16	−0.08	0.009	0.03	0.51
**AOD750**_μm_	0.20	<0.001	0.07	0.04	0.06	0.22	−0.12	<0.001	−0.02	0.61
**TISA500**_μm_	0.18	<0.001	0.10	0.005	0.05	0.20	−0.06	0.03	0.04	0.30
**TISA750**_μm_	0.22	<0.001	0.08	0.03	0.05	0.28	−0.10	0.003	0.004	0.92
**PIT500**_μm_	0.04	0.24	0.03	0.56	−0.06	0.45	−0.03	0.55	−0.05	0.07
**PIT750**_μm_	0.03	0.43	−0.01	0.90	−0.04	0.61	−0.02	0.65	−0.04	0.13
**PCT500**_μm_	0.08	0.15	0.01	0.77	−0.06	0.17	0.05	0.27	−0.24	<0.001
**PCT750**_μm_	0.14	0.01	−0.004	0.93	−0.08	0.09	0.11	0.03	−0.20	<0.001

**Table 4:** Summary of previous studies on anterior segment structures in pediatric eyes.

Study	Current Study	Edawaji B. S. A. et al. [11]	Daniel M. C. et al. [20]	Hashemi H. et al. [21]	Yeter V. et al. [22]
**Subject number**	709	223	50	683	110
**Age (years)**	8	2 day–15	4–16	6–18	3–6
**Published Year**		2021	2018	2015	2015
**Modality**	AS-OCT	Handheld OCT	AS-OCT	Optical	UBM
**AL**	available	NA	NA	available	available
**SE**	available	available	available	NA	available
**ACA**	available	NA	NA	NA	available
**AOD**	available	available	available	NA	NA
**TISA**	available	available	available	NA	NA
**PIT**	available	NA	NA	NA	NA
**PCT**	available	NA	NA	NA	available
**CCT**	NA	available	available	available	available
**Remarks**		trabecular meshwork length	trabecular meshwork length and height	central ACD	peripheral ACD

## Data Availability

Data are unsuitable for public deposition due to ethical restrictions and the legal framework of Japan. It is prohibited by the Act on the Protection of Personal Information (Act No.57 of 30 May 2003, amendment on 9 September 2015) to publicly deposit the data containing personal information. Ethical Guidelines for Medical and Health Research Involving Human Subjects enforced by the Japan Ministry of Education, Culture, Sports, Science and Technology and the Ministry of Health, Labour and Welfare also restricts the open sharing of the epidemiologic data. All inquiries about access to data should be sent to: rshinohara@yamanashi.ac.jp. The person responsible for handling enquiries sent to this e-mail address is Dr Ryoji Shinohara, director of Koshin Unit Center for JECS.
